# Exploring glymphatic system alterations in iRBD and Parkinson’s disease using automated DTI-ALPS analysis

**DOI:** 10.1038/s41531-025-00921-4

**Published:** 2025-04-15

**Authors:** S. Marecek, V. Rottova, J. Nepozitek, T. Krajca, R. Krupicka, J. Keller, D. Zogala, J. Trnka, K. Sonka, E. Ruzicka, P. Dusek

**Affiliations:** 1 Department of Neurology and Center of Clinical Neuroscience, First Faculty of Medicine, Charles University and General University Hospital in Prague, Prague, Czech Republic; 2 Czech Technical University in Prague, Faculty of Biomedical Engineering, Kladno, Czech Republic; 3 Department of Radiodiagnostics, Na Homolce Hospital, Prague, Czech Republic; 4 Institute of Nuclear Medicine, First Faculty of Medicine, Charles University and General University Hospital in Prague, Prague, Czech Republic

**Keywords:** Neuroscience, Biomarkers, Neurology

## Abstract

Diffusion tensor image analysis along the perivascular space (DTI-ALPS) is a potential non-invasive marker of glymphatic function that typically relies on manual region of interest (ROI) placement. This study compared ALPS indices in treatment-naïve, de novo diagnosed patients with Parkinson’s disease (PD), patients with isolated REM behavior disorder (iRBD), and healthy controls using both manual and automatic approaches to the ROI selection used in ALPS-index calculation. ALPS indices were analyzed bilaterally and correlated with clinical severity (MDS-UPDRS) and nigrostriatal denervation (DAT-SPECT). ANCOVA revealed significant inter-group differences using both manual (*p* = 0.018) and automatic (*p* = 0.002) ROI selection methods. The automatic ROI selection approach showed significantly lower ALPS indices in PD compared to controls (*p* = 0.001) and iRBD (*p* = 0.009). ALPS indices correlated with symptom severity and nigrostriatal denervation. These findings underscore the reliability of the automatic ROI placement approach for ALPS index calculation and may indicate early glymphatic alterations in Parkinson’s disease.

## Introduction

The glymphatic system is a whole-brain perivascular network that utilizes periarterial cerebrospinal fluid (CSF) influx, with its subsequent diffusion into the brain parenchyma and perivenous efflux to drive interstitial solute clearance^1,2^. Dysfunction of the glymphatic system is increasingly recognized as a contributing factor in several neurological disorders, including Parkinson’s disease (PD), normal pressure hydrocephalus, Alzheimer’s disease, and others^3–6^. The pathophysiological mechanisms of glymphatic dysfunction in PD are not well known. However, one study in mice has shown α-synuclein injection into basal ganglia caused delayed dural lymphatic vessel drainage, and vice versa, the ligation of draining lymphatic vessels caused increased α-synuclein accumulation and exacerbated motor and memory deficits^7^. In PD, glymphatic dysfunction has been observed even in the earliest stages of neurodegeneration, including its prodromal stages, represented by isolated rapid-eye-movement behavior disorder (iRBD)^8,9^.

RBD is a disorder characterized by dream enactment and loss of muscle atonia during REM sleep. It can be caused by narcolepsy, antidepressant intake, brainstem lesions; when a precise cause has not been identified, it is termed ‘isolated’ RBD. This isolated RBD (iRBD) is predominantly caused by early-stage synucleinopathies, with studies showing an approximate 70% conversion rate over 12 years to either PD, dementia with Lewy bodies, or multiple system atrophy^10–12^. This makes iRBD a valuable target for studying PD’s pathophysiology, course, and possible therapy^13,14^.

There are several potential ways to measure the function of the glymphatic system^15^, with diffusion tensor imaging along perivascular spaces (DTI-ALPS)—first described in 2017 by Taoka et al.^16^—being one of them. This method employs widely available MRI sequences and relies on the placement of regions of interest (ROI) next to the top of the lateral ventricles, into the areas of projection and association fibers. In these areas, the glymphatic flow traverses the perivascular spaces, perpendicular to the lateral ventricles and both the projection and association fibers. Diffusion tensor imaging can be used to estimate the movement of water along these perivascular spaces, theoretically reflecting the functionality of the glymphatic system^6^. However, one of the main critiques of the DTI-ALPS method is the lack of rigorous pathophysiological studies demonstrating its relationship to glymphatic function^6^. Nevertheless, one study reported a good correlation between glymphatic system functionality, as measured by intrathecal gadolinium injection, and the DTI-ALPS method^17^.

To the best of our knowledge, only two studies simultaneously compared patients with PD, iRBD, and healthy controls^9,18^. In one of these studies, iRBD patients were diagnosed based on history rather than polysomnography, classifying them as “possible” iRBD cases^18^. In the other study, each group was limited to only 20 participants^9^. In our study, we aim to compare the glymphatic function—as measured by DTI-ALPS—in newly diagnosed treatment naïve patients with PD, patients with video-polysomnography-confirmed iRBD and healthy controls. Additionally, we explore the relationship between the ALPS-index, clinical scores and nigrostriatal dopaminergic denervation measured by dopamine transporter single-photon emission computed tomography (DAT-SPECT). Furthermore, most of the studies using the DTI-ALPS method employ a manual selection of regions of interest (ROIs)^6^. This introduces the possibility of human error or bias. We aimed to employ an automatic ROI selection algorithm that would forego the need for selection of these ROIs by clinicians.

## Results

### Subject characteristics

Our study cohort initially comprised 91 patients with PD, 68 patients with iRBD, and 50 control subjects. Of these, 12 subjects were excluded due to preprocessing failures—specifically, failures during the *topup* step and the subsequent transformation to normalized space. One patient was excluded owing to a structural abnormality, and an additional 12 subjects were removed because an eccentric head position precluded the identification of manual regions of interest (ROIs). In total, 25 subjects were excluded, yielding a final sample of 79 PD patients, 57 iRBD patients, and 48 controls (Table 1).

**Table 1:** Comparison of demographic and clinical characteristics, and of ALPS indices in PD, iRBD, and controls

Characteristics	Controls (*n* = 48)	iRBD (*n* = 57)	PD (*n* = 79)	Inter-group *p* value	Partial *η*^2^	Between groups *p* values		
						PD vs. HC	PD vs. iRBD	iRBD vs. HC
Male sex (%)	34 (71)	49 (86)	48 (61)	**0.006**	–	n.s.	**0.001**	n.s.
Age (years)	61.5 ± 9.9	66.5 ± 7.2	59.5 ± 12.0	**<0.001**	–	n.s.	**<0.001**	**0.013**
MoCA	25.6 ± 2.4	23.7 ± 2.8	24.7 ± 3.1	0.031*	–	n.s.	n.s.	**0.009**
MDS-UPDRS I	3.0 ± 2.7	7.5 ± 5.3	5.8 ± 4.3	<0.001*	–	**0.001**	**0.049**	**<0.001**
MDS-UPDRS II	0.7 ± 1.5	2.6 ± 3.9	7.5 ± 4.9	<0.001*	–	**<0.001**	**<0.001**	**0.016**
MDS-UPDRS III	3.4 ± 4.0	6.1 ± 5.4	30.3 ± 13.1	<0.001*	–	**<0.001**	**<0.001**	n.s.
Disease duration**	–	8.2 ± 8.5	2.0 ± 1.8	–	–	–	–	–
*ALPS indices calculated using manually selected ROIs*								
Average	1.35 ± 0.17	1.31 ± 0.16	1.28 ± 0.19	0.018*	0.043	**0.008**	n.s.	n.s.
Left hemisphere	1.31 ± 0.16	1.27 ± 0.17	1.26 ± 0.20	n.s.*	–	–	–	–
Right hemisphere	1.40 ± 0.23	1.35 ± 0.18	1.31 ± 0.20	0.009*	0.051	**0.004**	**0.028**	n.s.
*ALPS indices calculated using automatically selected ROIs*								
Average	1.28 ± 0.11	1.24 ± 0.14	1.21 ± 0.18	0.002*	0.067	**0.001**	**0.009**	n.s.
Left hemisphere	1.29 ± 0.11	1.25 ± 0.14	1.22 ± 0.19	0.003*	0.063	**0.001**	**0.018**	n.s.
Right hemisphere	1.27 ± 0.13	1.24 ± 0.14	1.20 ± 0.18	0.003*	0.061	**0.003**	**0.009**	n.s.

Where applicable, the data are presented as mean ± standard deviation, with ALPS indices adjusted for age.

*MoCA* Montreal Cognitive Assessment, *MDS-UPDRS* Movement Disorder Society-Sponsored Revision of the Unified Parkinson’s Disease Rating Scale, *HC* healthy controls, n.s. not significant, bold for *p* < 0.05.

*Adjusted for age and sex.

**Disease duration is defined as the subjective duration of dream enactment behavior in iRBD and time since the occurrence of the first motor symptom in PD.

The iRBD group was significantly older than both the PD and control groups, comprised a higher proportion of males than the PD group, and exhibited lower MoCA scores compared to the control group. Notably, the MoCA scores remained significantly lower in the iRBD group relative to controls even after controlling for age and sex (*p* = 0.009).

The PD group demonstrated significantly higher MDS-UPDRS parts II and III scores than both the iRBD and control groups, and significantly higher MDS-UPDRS part I scores than the control group. Additionally, the iRBD group showed significantly higher MDS-UPDRS parts I and II scores compared to the control group and higher MDS-UPDRS part I scores than the PD group.

### Reliability analysis of manual and automatic ROI selection approaches

To evaluate the reliability of the ALPS indices derived from our ROI selection approaches—manual and automatic—we compared the three ALPS indices (average, left-hemisphere, and right-hemisphere) obtained by each approach.

ALPS indices calculated via manual ROI selection were significantly higher than those obtained using the automatic approach (manual: mean = 1.30, SD = 0.18; automatic: mean = 1.23, SD = 0.16; *t*(183) = 9.334, *p* < 0.001, Cohen’s *d* = 0.41). This significant difference was also observed for both the left-hemisphere (*p* = 0.001) and right-hemisphere (*p* < 0.001) ALPS indices.

**Fig. 1:**
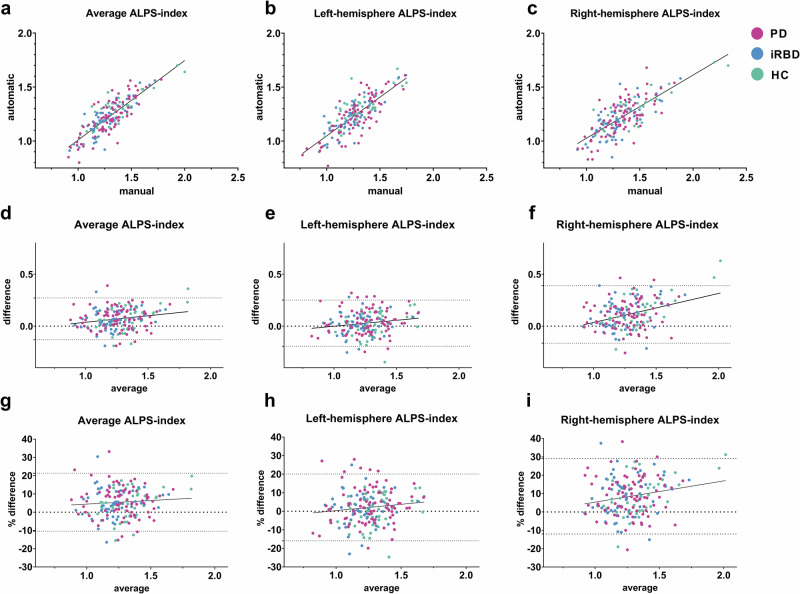
Analysis of differences in manual and automatic approaches to ROI selection used in ALPS-index calculation. Scatter plots representing the relation of manual and automatic ROI selection approaches (**a**–**c**). Bland–Altman plots using the numeric difference values (**d**–**f**) and % difference values (**g**–**I**) between the manual and automatic approaches.

A strong association was found between the ALPS indices obtained from the two ROI selection approaches: for the average ALPS index, *r*(182) = 0.83 (*p* < 0.001); for the left-hemisphere ALPS-index, *r*(182) = 0.80 (*p* < 0.001); and for the right-hemisphere ALPS-index, *r*(182) = 0.75 (*p* < 0.001). Scatter plots illustrating these relationships are presented in Fig. 1.

Bland–Altman plots were generated using the numeric differences between the ALPS indices from the manual and automatic methods (Fig. 1). In these plots, fewer than 10% of subjects fell outside the 95% limits of agreement. As the values of all three ALPS indices increased, a significant (*p* < 0.05) rise in the absolute differences between the automatic and manual ROI selection approaches was observed. When differences were expressed as percentages, this relationship was maintained for the left- and right-hemisphere ALPS indices, though not for the average ALPS-index.

### Inter-group differences in ALPS indices

**Fig. 2:**
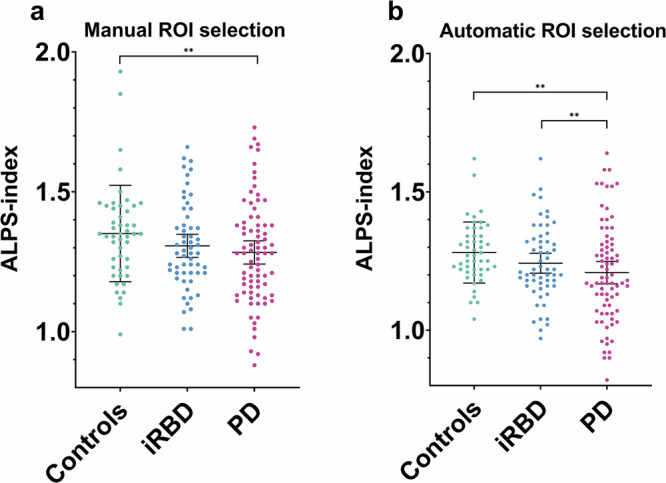
Differences in ALPS indices between PD, iRBD, and HCs. Scatter plots displaying differences in ALPS indices calculated using the manual (**a**) and automatic (**b**) ROI selection methods in PD, iRBD, and HCs. Volumes are adjusted for age, and the lines and whiskers represent the mean and standard deviations, respectively. **for *p* < 0.01.

When comparing side-averaged ALPS indices, we found an inter-group difference using both the manual [*F*(2,180) = 4.081, *p* = 0.018] and the automatic [*F*(2,180) = 6.448, *p* = 0.002] ROI selection approaches. In the post-hoc analysis, PD subjects had lower ALPS indices using both the manual [*p* = 0.008, *p* = 0.051 (PD vs. controls, PD vs. iRBD)] and automatic [*p* = 0.001, *p* = 0.009 (PD vs. controls, PD vs. iRBD)] ROI selection approaches (Fig. 2, Table 1).

The results in left- and right-sided age-adjusted ALPS indices were comparable to the average values, except for the left-sided ALPS indices calculated via the manual ROI selection approach, where no inter-group difference was found (Table 1).

As the iRBD group was significantly older than the PD group and had significantly more male participants, we performed a sensitivity analysis in age- and sex-matched subgroups. This yielded 40 participants in each group. Using identical methods, we found an inter-group difference in the automatic approach [*F*(2,116) = 5.607, *p* = 0.005], with the PD group having significantly lower ALPS indices [*p* = 0.003, *p* = 0.007 (PD vs. controls, PD vs. iRBD)]. We did not find any significant inter-group differences using the manual approach (*p* = 0.067).

### Effects of nigrostriatal denervation

**Fig. 3:**
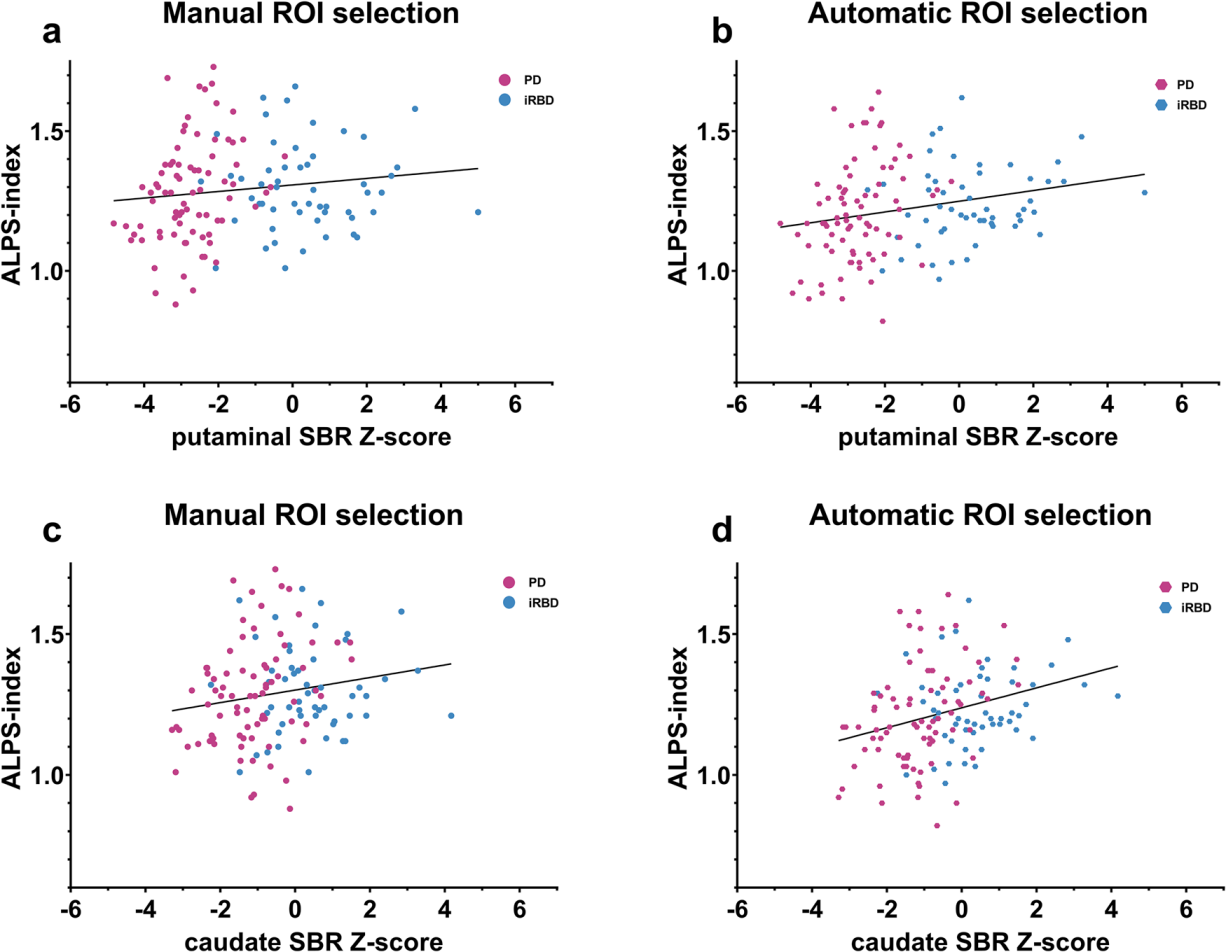
Average putaminal and caudate SBR *Z*-scores in relation to ALPS indices. Scatter plots illustrating the relationship between average putaminal and caudate SBR *Z*-scores and ALPS indices calculated using both the manual and automatic ROI selection approaches. Panel **a** shows the manual approach for putaminal SBR *Z*-scores, panel **b** the automatic approach for putaminal SBR *Z*-scores, panel **c** the manual approach for caudate SBR *Z*-scores, and panel **d** the automatic approach for caudate SBR *Z*-scores. Volumes adjusted to age. PD and iRBD subjects are pooled.

We found a positive correlation between ALPS indices and mean putaminal SBR *Z*-scores using both the manual [*p* = 0.029, *r*(129) = 0.191] and automatic [*p* < 0.001, *r*(129) = 0.310] approaches in the PD-iRBD pooled group (Fig. 3). When analyzing the PD group separately, the correlation remained significant for both the manual [*p* = 0.027, *r*(76) = 0.250] and automatic [*p* = 0.011, *r*(76) = 0.286] approaches.

Similar results were observed for caudate SBR *Z*-scores. In the PD-iRBD pooled group, ALPS indices calculated using ROIs selected by the automatic method showed a significant correlation [*p* < 0.001, *r*(129) = 0.306]; however, the manual selection method did not yield a significant correlation (*p* = 0.051)(Fig. 3). When analyzing the PD group separately, the correlation remained significant only for the automatic approach [*p* = 0.039, *r*(76) = 0.234] but not for the manual approach (*p* = 0.211).

We further compared ALPS indices between the most affected and least affected hemispheres with respect to putaminal and caudate SBR *Z*-scores on DAT-SPECT. No significant differences were found between the most and least affected hemispheres in the pooled group or when analyzing the PD and iRBD groups separately.

### Associations with clinical features

Several negative correlations were found between ALPS indices and MDS-UPDRS scores in patients with PD (Table 2). Specifically, the manual ROI selection approach was correlated with the MDS-UPDRS I score, while both manual and automated ROI selection approaches were correlated with the MDS-UPDRS II and III scores. Additionally, ALPS indices were correlated with the bradykinesia and axial subscores of the MDS-UPDRS III in PD.

**Table 2:** Associations between ALPS indices calculated using manual and automatic ROI selection approaches and symptom severity in Parkinson’s disease

	MDS-UPDRS I	MDS-UPDRS II	MDS-UPDRS III	Tremor	Rigidity	Bradykinesia	Axial
Manual	**0.005(−0.32)**	**<0.001(−0.45)**	**0.002(−0.35)**	0.665(−0.05)	0.135(−0.172)	**<0.001(−0.41)**	**0.003(−0.34)**
Automatic	0.055**(**−0.22)	**0.001(−0.36)**	**0.028(−0.25)**	0.626(0.06)	0.220(−0.14)	**0.006(−0.31)**	**0.001(−0.37)**

Using two-tailed partial correlation on age-adjusted ALPS indices, controlling for age and sex Values presented as *p*-value (partial correlation coefficient). Bold for *p* < 0.05.

In contrast, no significant correlations were observed between age-adjusted ALPS indices and MoCA scores in any of the groups.

## Discussion

Our results show lower ALPS indices—indicating decreased diffusion in the direction of glymphatic flow—in patients with PD compared to patients with iRBD and healthy controls. Moreover, this decrease correlates with nigrostriatal denervation, as measured by DAT-SPECT, and clinical severity, as measured by MDS-UPDRS clinical scales.

The DTI-ALPS method, first described by Taoka et al. in 2017^16^, is a relatively new approach for measuring glymphatic system dysfunction. As such, the method still lacks rigorous pathological verification^6,19^ to prove that ALPS-index represents the actual magnitude of glymphatic flow. However, at least one study did show a correlation with glymphatic system function as measured by intrathecal application of gadolinium^17^.

Another critique of the DTI-ALPS method is the possibility of human error during the ROI selection process. We addressed this issue by incorporating an automatic ROI selection approach, which, based on our findings, demonstrated performance comparable to or superior to that of the manual method. Our approach was similar to other studies that employed an atlas-based method for ROI selection—one study used a similar JHU atlas^20^, while others used the ICBM DTI-81 atlas^4,21^.

When comparing the manual and automatic approaches for ROI selection, we found that ALPS indices calculated using the manual approach were significantly higher than those obtained via automatic ROI selection. Bland-Altman plots further revealed that the difference between the methods increased with higher ALPS indices, although only a small number of subjects fell outside the 95% limits of agreement. Nevertheless, the correlation between the methods was excellent.

In our study, all ALPS indices (average, left, right, calculated using both manual and automatic ROI selection approaches) showed similar results, except the left-hemisphere ALPS indices calculated using the manual approach, which did not show significant between-group differences. This finding is unexpected, as the placement of left-sided association ROIs is generally easier since the superior longitudinal fasciculus of right-handed subjects is better discernible on the usually dominant left side (as most of the population is right-handed)^6^. This could be due to human error during ROI selection, which highlights the need for automated ROI selection. Future studies should verify the performance of different atlases in automatic DTI-ALPS calculation.

We found two previous studies comparing ALPS indices in PD, iRBD, and healthy control subjects. Both found significant differences in ALPS indices between iRBD and control subjects. One study included “probable” iRBD (cases not confirmed by polysomnography) and found significant differences between all three groups^18^. The other study included iRBD subjects confirmed by polysomnography and found significant differences between iRBD and HC, but not between iRBD and PD subjects; however, it included only 20 patients in each group^9^. Some previous studies compared ALPS indices in iRBD and control subjects only and found significantly reduced ALPS indices in iRBD subjects^8^. In our study, we did not confirm this finding. This could be due to several reasons, including inter-rater variability in the selection of manual ROIs used in these studies, differences in patient populations, and a relatively low number of iRBD patients, which may have led to limited statistical power. Our iRBD subjects were recruited “on-demand” by a media campaign. This difference—in that patients were approached rather than spontaneously seeking medical help for their iRBD symptoms—could, in our case, select for a different, perhaps earlier-stage, patient population than in previous studies. This is supported by a study by Joza et al.^22^, which found that self-referral through outreach initiatives was associated with earlier clinical presentation and subsequent slower progression.

We found two studies comparing ALPS indices to DAT-SPECT. One study involved drug-naïve PD patients, while the other focused on individuals with iRBD, neither finding a correlation between ALPS indices and DAT-SPECT^9,23^. Another study found a correlation between nigral dopaminergic denervation and ALPS indices using a hybrid 18F-fluorodopa PET-MRI^24^. Even when employing a simpler approach to nigrostriatal denervation imaging, our results indicate that ALPS indices are significantly correlated with the degree of nigrostriatal denervation in PD patients—an association not observed in iRBD patients.

The absence of significant differences in ALPS indices between iRBD patients and healthy controls, together with the observed correlations between ALPS indices, motor impairment (as measured by UPDRS II and III scores), and nigrostriatal denervation in PD, suggests that glymphatic dysfunction may be a dynamic process that worsens with disease progression. However, as the DTI-ALPS method lacks pathological validation, further verification is needed—either of the DTI-ALPS method itself or by using other methods of measuring glymphatic dysfunction. Future studies will also be needed to assess the extent to which glymphatic system dysfunction is accelerated by synucleinopathy, compounding the “physiological” age-related decline in glymphatic flow.

An interesting finding is the correlation of ALPS indices with MDS-UPDRS scores and bradykinesia and axial MDS-UPDRS III subscores. The correlation with MDS-UPDRS scores has been described before^23,25^. In a previous study, the results differed, with ALPS indices correlating with only the rigidity subscores^26^.

Our study did not find a correlation between ALPS indices and MoCA. There are inconsistent results regarding the correlation between cognitive dysfunction and ALPS indices. Some studies did not find a correlation between ALPS indices and MoCA^18,26^, whereas others described a relationship between cognitive dysfunction and ALPS indices^5^. However, our subjects were in a very early stage of the disease. As overt cognitive deficit is not a feature of iRBD or generally of early-stage PD, it is possible that the association would appear at later stages, when a larger spread of MoCA scores might be observed due to the onset of dementia in a subset of patients.

The finding of significantly higher rates of non-motor signs (as measured by UPDRS Part I) in iRBD patients compared to both PD and HC subjects was previously described in a paper by Barber et al.^27^, in which an analysis of a large cohort of iRBD and de novo PD patients showed similar results. In that study, the iRBD patients exhibited higher scores for depression, apathy, and anxiety compared to those with established PD. A possible explanation given was that PD patients who progress from iRBD tend to exhibit the akinetic-rigid/postural instability-gait difficulty subtype^28^, which is associated with a more severe non-motor phenotype^29^. This is also in line with the observation that iRBD relates to the “diffuse malignant” or “body first” subtype of PD as well as DLB, which is characterized by higher rates of non-motor signs and mild cognitive impairment^30,31^. Indeed, unlike the PD group, iRBD patients in this study had significantly lower MoCA scores than healthy controls.

The higher age observed in the iRBD group may be explained by our unselected PD cohort, which includes a proportion of early-onset patients. Multicenter studies indicate that the age of onset for iRBD is generally later than that for unselected PD; for instance, approximately 8% of iRBD patients are younger than 55 years^12^, compared to about 27% of PD patients being younger than 56 years^32^. Moreover, the mean ages reported in these multicenter studies—66.3 years for iRBD and 61.7 years for PD—are roughly in line with the demographic patterns observed in our cohorts.

We also did not find a side difference between the more affected side and the less affected side by nigrostriatal denervation. This could be explained by the glymphatic dysfunction being a global process affected by overall neurodegeneration rather than by “local” side differences in nigrostriatal denervation.

Fluid flow through the glymphatic system is inextricably linked with sleep^33^. In the case of the DTI-ALPS method, one study found a connection between sleep disruption—as measured by the Pittsburgh Sleep Quality Index—and decreased ALPS indices^34^. Another study found that ALPS indices were negatively correlated with blood levels of neurofilament light chain in patients with traumatic brain injury^35^. In the context of neurodegeneration, a study by Xie et al.^36^ described a 95% decrease in tracer influx in awake mice compared to sleeping mice, accompanied by a two-fold increase in Aβ clearance in sleeping mice. This implies a connection between sleep, glymphatic system function, and proteinopathies^33,37^. However, although sleep disturbances are common in PD^38^ and patients with PD show impaired meningeal lymphatic function^7^, the precise pathophysiological connections remain to be explored.

In conclusion, our results show a decrease in ALPS indices in PD compared to iRBD and HCs, which could be a sign of glymphatic dysfunction in PD. The ALPS indices in PD correlated with clinical severity as measured by MDS-UPDRS scores and nigrostriatal denervation as measured by DAT-SPECT. This could suggest that glymphatic dysfunction is a dynamic process that worsens with disease progression. The ALPS indices of iRBD patients were numerically intermediate between those of the PD and HC groups, with significant differences observed only between iRBD and PD patients. Moreover, we have shown that an automatic approach to ROI selection is comparable, and in some cases even superior, to the manual approach.

## Methods

### Participants

Our subject population consisted of de novo diagnosed treatment-naïve patients with PD, iRBD patients, and healthy controls recruited at the Department of Neurology, First Faculty of Medicine, Charles University and General University Hospital in Prague from 2015 to 2021. The PD patients were part of the BIO-PD cohort described previously^39^ and were diagnosed according to the Movement Disorders Society (MDS) clinical diagnostic criteria^40^. The iRBD patients were diagnosed in accordance with the International Classification of Sleep Disorders, third edition (ICSD-3) using video-polysomnography^41^. Patients with RBD secondary to focal brainstem lesions, narcolepsy, medication usage, and clinically manifest dementia or parkinsonism, were excluded. The control subjects were recruited from the general community via advertisements. Eligibility criteria included the absence of active oncologic illness, significant neurological disorders, and abuse of psychoactive substances. RBD was excluded in all control subjects by history and video-polysomnography. All study participants were examined according to a complex protocol including neurological examination, structured interview, Montreal Cognitive Assessment (MoCA)^42^, and MDS-sponsored Revision of the Unified Parkinson’s Disease Rating Scale (MDS-UPDRS)^43^. The study was approved by the Ethics Committee of the General University Hospital in Prague (IORG0002175, IRB00002705, FWA00029052). Participants signed informed consent before entering the study, in accordance with the Declaration of Helsinki.

### Imaging acquisition protocol

MRI examination was performed on a 3 T scanner (Siemens Skyra 3 T, Siemens Healthcare, Erlangen, Germany) with a 32-channel head coil. The protocol included diffusion tensor MRI with repetition time (TR) = 10.5 s; echo time (TE) = 93 ms; total 72 slices with isotropic voxel resolution of 2 mm; 30 noncolinear directions with *b*-value of 1000 s/m^2^ and one *b* = 0 s/m^2^ image in the anterior–posterior and posterior–anterior phase encoding directions; and an axial 3D T1-weighted Magnetization Prepared Rapid Gradient Echo (MPRAGE, TR 2200 ms; TE 2.4 ms; inversion time (TI) 900 ms; flip angle (FA) 8°; field of view (FOV) 230 × 197 × 176 mm; isotropic voxel resolution 1 mm).

In all PD patients and all but four iRBD patients, DAT-SPECT was performed using the [123I]-2-b-carbomethoxy-3b-(4-iodophenyl)-N-(3-fluoropropyl) nortropane ([123I]FP-CIT, DaTscan®, GE Healthcare, Little Chalfont, Buckinghamshire, UK) tracer according to European Association of Nuclear Medicine (EANM) procedure guidelines^44^; the detailed protocol is described elsewhere^45^. Automated semi-quantitative analysis was performed using the DaTQUANT v. 2.0 software (GE Healthcare, USA), and the *Z*-scores of specific binding ratios (SBR) in both putamina and caudate relative to background binding were calculated. DAT-SPECT was performed within one month of MRI. All PD patients were scanned before the introduction of dopaminergic therapy.

### Calculating the DTI-ALPS index

For general image preprocessing, we employed FSL^46,47^ and MRtrix3^48,49^. An automatic processing pipeline was developed using Snakemake^50,51^, with the most computationally intensive tasks executed on the CESNET MetaCentrum distributed computing infrastructure. All analyses were performed in each subject’s native diffusion space. The DWI data were denoised, corrected for Gibbs ringing artifacts, and adjusted for distortions, eddy currents, and head movement. Subsequently, FSL’s DTIFIT tool was used to generate the *Dxx*, *Dyy*, and *Dzz* diffusivity images.

The DTI-ALPS method is predicated on the placement of regions of interest (ROIs) within the white matter areas of the association and projection fibers adjacent to the apex of the lateral ventricles. In these regions, the perivascular spaces—along which glymphatic flow is oriented—run along the *x*-axis, while association fibers extend along the *y*-axis and projection fibers along the *z*-axis. The ALPS-index is calculated as the ratio of the mean diffusivity along the *x*-axis (which includes the glymphatic flow) to the mean diffusivity along the axes lacking both glymphatic flow and white matter fibers (specifically, the *y*-axis in the projection area and the *z*-axis in the association area), as expressed in Eq. (1):

1 $$\begin{array}{l}{\rm{ALPS}}-{\rm{index}}={\rm{mean}}({\mathrm{Dxx}}\,{\rm{projection}},\,{\mathrm{Dxx}}\,{\rm{association}})\\\qquad\qquad\qquad\quad/{\rm{mean}}({\mathrm{Dyy}}\,{\rm{projection}},\,{\mathrm{Dzz}}\,{\rm{association}})\end{array}$$

**Fig. 4:**
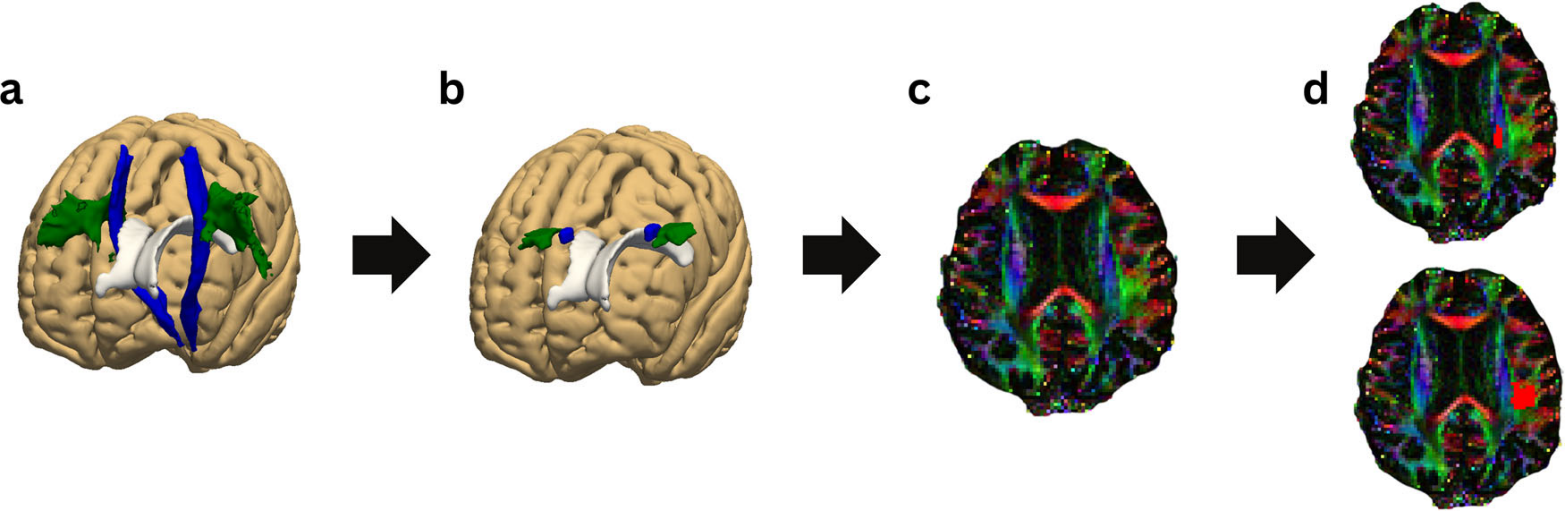
Automatic ALPS ROI selection. We used the “JHU ICBM tracts maxprob thr25 1 mm” atlas labels of superior longitudinal fasciculus and corticospinal tract, for the association and projection areas, respectively (**a**). We restricted these labels to the area on the top of the lateral ventricles and excluded parts close to the cortex (**b**). We then calculated a warp from the MNI152 space to each subject’s diffusion space (**c**) and transformed the masks (**d**). The images presented in steps **c** and **d** display fractional anisotropy-modulated diffusivity in the *x* (red), *y* (green), and *z* (blue) directions.

Two methods of ROI selection were employed: manual and automatic. In the manual approach, a blinded rater (V.R.) placed cubic ROIs measuring 6 × 6 × 6 mm within the projection and association white matter regions of both hemispheres. For the automatic approach (Fig. 4), the labels from the “JHU ICBM tracts maxprob thr25 1 mm” atlas corresponding to the superior longitudinal fasciculus (SLF) and corticospinal tract (CST) was used for the association and projection regions, respectively^52–54^. These labels were restricted to the region at the apex of the lateral ventricles, with portions adjacent to the cortex excluded to prevent potential intrusion during the transformation to each subject’s diffusion space. A single transformation from MNI-152 space to each subject’s diffusion space was generated using FSL via a three-step process: (1) FLIRT’s linear transformation registered each subject’s *b* = 0 diffusion image to their structural T1 image; (2) FLIRT and FNIRT’s non-linear transformations registered the T1 images to the MNI-152 template; and (3) CONVERTWARP produced a unified transformation from the MNI-152 template to each subject’s diffusion space. Finally, the restricted SLF and CST ROIs were transformed into each subject’s diffusion space and binarized, yielding the association and projection ROI masks used in the ALPS-index calculation.

### Statistics

Statistical analyses were performed using IBM SPSS for Windows (Version 26). The chi-square test was employed to compare the sex distribution between groups, and a one-way analysis of variance (ANOVA) was applied to assess inter-group differences in age. A one-way analysis of covariance (ANCOVA), with age and sex as covariates, was used to evaluate differences in MDS-UPDRS and MoCA scores. Given the known association between the ALPS-index and age^55^, ALPS indices were adjusted for age using the regression coefficient (β) and the mean age from control subjects as described in Eq. (2)^56^:

2 $${\mathrm{ALPS}-\mathrm{index}}_{{\mathrm{adjusted}}\,{\mathrm{i}}}={\mathrm{ALPS}}-{\mathrm{index}}_{{\mathrm{raw}}\,{\mathrm{i}}}-\upbeta\,\left({\mathrm{Age}}_{{\mathrm{raw}}\,{\mathrm{i}}}-{\mathrm{Age}}_{\mathrm{mean}}\right)$$

Subsequently, a one-way ANCOVA with sex as a covariate was used to assess inter-group differences in the age-adjusted ALPS indices. All post-hoc multiple comparisons were conducted using the least significant difference (LSD) method, and effect sizes were expressed as partial eta squared (partial *η*²).

Partial correlation analyses—with sex as a covariate—were conducted to examine the relationship between age-adjusted ALPS indices and the average putaminal and caudate SBR *Z*-scores from both hemispheres. Additionally, age and sex were included as covariates when investigating correlations with MoCA and MDS-UPDRS scores. This method was also applied to evaluate the correlations between ALPS indices and the following MDS-UPDRS III subscores: tremor (sum of items 15–18), bradykinesia (sum of items 2, 4–9, and 14), rigidity (item 3), and axial (sum of items 1 and 9–13) subscores^57^.

To assess potential side differences in ALPS indices relative to the degree of nigrostriatal denervation, the paired Student’s *t*-test was used to compare the side with the higher putaminal SBR *Z*-score against the side with the lower putaminal SBR *Z*-score on DAT-SPECT. The same approach was used for caudate SBR *Z*-scores.

Sensitivity analyses were performed using age- and sex-matched subgroups to mitigate potential bias. Matching was conducted using SPSS’s case-control matching utility with a randomized case order for match selection and a tolerance value of 8 years for age and 0 for sex.

Finally, to compare the manual and automatic approaches across all parameters (i.e., average, right-hemisphere, and left-hemisphere ALPS indices), three statistical methods were employed: (1) a paired Student’s *t*-test to assess significant differences; (2) bivariate correlation analysis using the Pearson correlation coefficient; and (3) Bland–Altman plots to evaluate agreement based on both numeric and percentage differences between the methods. Additionally, linear regression was performed to investigate the relationship between average ALPS-index values and the differences (both numeric and percentage) between ALPS indices calculated using the manual and automatic ROI selection approaches.

## Data Availability

The datasets used and analyzed during the current study are available from the corresponding author on request.
